# Interaction of Lead(II) Perchlorate with *N*′‑Isonicotinoylpicolinohydrazonamide and Its Sodium Salt in the Presence of Potassium Cyanide: Yellow Green Light Emitting Phosphors, Stabilized by Tetrel Bonds, and a System to Transform Methanol to Acetate

**DOI:** 10.1021/acs.inorgchem.5c02419

**Published:** 2025-07-09

**Authors:** Ghodrat Mahmoudi, Isabel Garcia-Santos, Tamara Iglesias-Pereiro, Alfonso Castiñeiras, Julio Corredoira-Vázquez, Atash V. Gurbanov, Ennio Zangrando, Rosa M. Gomila, Antonio Frontera, Damir A. Safin

**Affiliations:** † Department of Chemistry, Faculty of Science, University of Maragheh, P.O. Box 55136-83111 Maragheh, Iran; ‡ Chemistry Department, Faculty of Engineering and Natural Sciences, Istinye University, Sarıyer, Istanbul 34396, Turkey; § Departamento de Química Inorgánica, Facultad de Farmacia, Universidad de Santiago de Compostela, 15782 Santiago de Compostela, Spain; ∥ Departamento de Química Inorgánica, Facultade de Química, Universidad de Santiago de Compostela, 15782 Santiago de Compostela, Spain; ⊥ Institute of Materials (iMATUS), Universidad de Santiago de Compostela, 15782 Santiago de Compostela, Spain; # Excellence Center, Baku State University, Z. Xalilov Str. 23, AZ 1148 Baku, Azerbaijan; ∇ Department of Chemical and Pharmaceutical Sciences, 9315 University of Trieste, Via L. Giorgieri 1, 34127 Trieste, Italy; ○ Departament de Química, 16745 Universitat de les Illes Balears, Crta de Valldemossa km 7.5, 07122 Palma de Mallorca (Baleares), Spain; ◆ Department of Chemistry, Universitat de les Illes Balears, 07122 Palma de Mallorca (Baleares), Spain; ¶ Scientific and Educational and Innovation Center for Chemical and Pharmaceutical Technologies, Ural Federal University named after the First President of Russia B.N. Yeltsin, Ekaterinburg 620002, Russian Federation; †† University of Tyumen, Volodarskogo Str. 6, 625003 Tyumen, Russian Federation

## Abstract

A coordination polymer is synthesized via the reaction of a mixture of Pb­(ClO_4_)_2_ and KCN with *N′*-isonicotinoylpicolinohydrazonamide (**HL**), leading to the formation and stabilization of a {[Pb_3_L_3_(OAc)­(H_2_O)]­(ClO_4_)_2_}*_n_* ·*n*H_2_O (**1**·*n*H_2_O) network, with the formation and trapping of the acetate anion. Using the sodium *N*-(amino­(pyridin-2-yl)­methylene)­isonicotinohydrazonate (**NaL**) instead of **HL** yields complex [Pb_2_L_2_OH]­(ClO_4_)·H_2_O (**2**·H_2_O) with the formation and trapping of the hydroxide anion. In **1**·*n*H_2_O, an almost linear aggregate is produced when the 4-pyridine fragments of the two [PbL]^+^ are coordinated to the metal cation of the third species, joining the three [PbL]^+^ cations. In **1**·*n*H_2_O, supramolecular stability is enhanced by Pb···O and Pb···π interactions involving the electron-rich core (Cg) of a ten-membered hydrogen-bonded ring, indicative of tetrel-type bonding. [Pb_2_L_2_OH]^+^ in **2**·H_2_O is formed, when a pair of the [PbL]^+^ cations are joined by a hydroxide anion. The Pb···N tetrel bonds bind [Pb_2_L_2_OH]^+^ to form a zigzag-like 1D supramolecular cationic chain. Both complexes are emissive in methanol. The emission profiles of **1**·*n*H_2_O and **2**·H_2_O in MeOH correspond to CIE-1931 chromaticity coordinates of (0.406, 0.492) and (0.420, 0.498), placing them within the yellow-green region of the visible spectrum. DFT studies are applied to reveal noncovalent interactions, including tetrel bonding, governing the supramolecular architectures. Electrostatic potential and QTAIM analyses confirm the presence of σ-holes at the Pb^2+^ cation in both structures.

## Graphical abstract

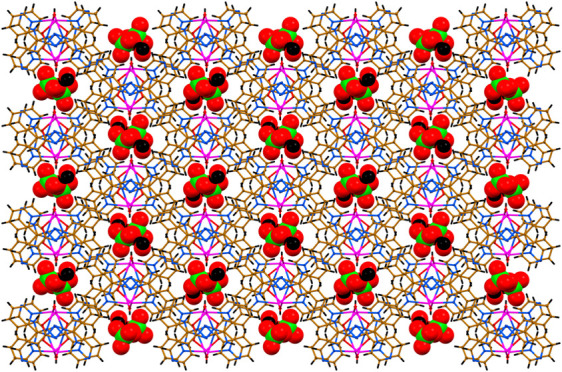

## 1 Introduction

The lead­(II) cation (Pb^2+^) is in coordination chemistry owing to its flexible coordination environment and relatively large ionic size. Moreover, distortions in the coordination sphere of Pb^2+^ complexes are likely attributed to the stereochemically active 6s^2^ lone pair.^1–6^ The so-called tetrel interactions often control the self-assembly of the Pb^2+^-based coordination polymers.^7–12^ Therefore, both organic ligands and counterions, as well as the emergence of supramolecular interactions, can affect the Pb^2+^-derived structures.^10,13–16^ The well-known chelating ligands based on pyridine-containing hydrazides can also couple with metal centers by means of their pyridine nitrogen atoms.

Since their discovery as cyanide-based inorganic chemicals almost 90 years ago, coordination polymers have been widely recognized.^17^ The first coordination organic ligand-derived polymer was announced approximately 65 years ago.^18^ Recent advancements in coordination polymers have garnered significant attention, particularly as metal–organic frameworks (MOFs), which were first conceptualized around three decades ago, continue to evolve as a focal point in the field.^19,20^ Coordination polymers consist of metal cations or metal-containing nodes bridged by organic ligands, forming extended coordination networks that organize into one-dimensional (chain), two-dimensional (network) or three-dimensional (framework) architectures.^21^

Poly-N-donor molecules appear to offer a highly versatile strategy for the design and synthesis of coordination polymer architectures..^22^ Furthermore, a Schiff base backbone is an additional appropriate structural unit for the production of coordination polymers.^23^ Therefore, incorporating both a Schiff base moiety and multiple nitrogen donor sites within a single ligand provides an efficient platform for assembling coordination polymers.

One of the effective methods for producing extended structures is noncovalent interactions. In this regard, tetrel bonding seems to be a potent tool for supramolecular structures.^24–60^ We have also been investigating the coordination behavior of Pb^2+^ ions, with particular attention to their ability to promote extended architectures through both coordination and tetrel-type noncovalent interactions.^9–12,61–83^ In the present contribution, we have directed our focus on the interaction of a mixture of Pb­(ClO_4_)_2_ and KCN with *N′*-isonicotinoylpicolinohydrazonamide (**HL**) and it sodium salt, namely sodium *N*-(amino­(pyridin-2-yl)­methylene)­isonicotinohydrazonate (**NaL**),^84^ which may act as a polydentate ligand. The isolated coordination compounds were studied using FTIR and ^1^H NMR spectroscopy. Structural elucidation was achieved by single-crystal X-ray diffraction analysis. UV–vis absorption and fluorescence measurements were used to investigate the optical characteristics of the complexes. DFT calculations were carried out on models of polymer **1** and a fragment of **2** to analyze the energetic features of tetrel bonding interactions, alongside other conventional noncovalent forces including aromatic π···π stacking and hydrogen bond interactions, which significantly contribute to the stabilization and organization of the crystal packing in both complexes.

## 2 Experimental Methods

### 2.1 Chemicals and Physical Measurements

All reagents were purchased from commercial suppliers and used as received, without further purification. FTIR spectra were acquired using KBr pellets on a PerkinElmer FT 801 spectrometer. ^1^H NMR spectra were collected in deuterated dimethyl sulfoxide solutions. Absorption, and emission and excitation spectral data were acquired from freshly prepared MeOH solutions (8 × 10^–5^ M) with a JASCO V-730 spectrophotometer and a SHIMADZU RF-6000 spectrofluorophotometer, respectively. Elemental analyses were performed using a LECO CHNS analyzer.

### 2.2 Synthetic Protocols

An aqueous solution (2 mL) of Pb­(ClO_4_)_2_·3H_2_O (38.2 mg, 0.083 mmol) and KCN (11.1 mg, 0.17 mmol) was mixed with a methanolic solution (30 mL) of **HL** or **NaL** (20.0 and 21.8 mg, respectively; 0.083 mmol). The reaction mixture was stirred for 30 min and subsequently allowed to stand undisturbed at ambient temperature for slow solvent evaporation, producing yellow crystals with a needle-like morphology over several days. **Caution!** *Although no incidents occurred during this study*, *perchlorate salts are potentially explosive and should always be handled with caution*, *using minimal quantities and appropriate safety protocols!*

**1**· ***n*H**_**2**_ **O**. Yield: 24 mg (53%). Anal. Calc. for C_38_H_37_Cl_2_N_15_O_15_Pb_3_ (1636.31): C 27.89, H 2.28 and N 12.84%; found: C 28.14, H 2.29 and N 12.98%.

**2**·**H**_**2**_ **O**. Yield: 32 mg (74%). Anal. Calc. for C_24_H_23_ClN_10_O_8_Pb_2_ (1029.36): C 28.00, H 2.25 and N 13.61%; found: C 28.39, H 2.01 and N 13.34%.

### 2.3 X-ray Diffraction Data Refinement

Single-crystal X-ray diffraction data were collected at 100(2) K using a Bruker APEX-II CCD diffractometer equipped with a graphite-monochromated Mo–Kα radiation source (λ = 0.71073 Å). Data integration and reduction were carried out using the APEX3 software suite,^85^ and absorption corrections were applied via the SADABS algorithm.^86^ The structure was solved by direct methods using the program SHELXS-97 ^87^ and refined through full-matrix least-squares minimization implemented in SHELXL-2019/2.^87^ All non-hydrogen atoms were refined with anisotropic displacement parameters and the hydrogen atoms were placed at calculated positions except those of NH_2_ groups which were refined. Crystallographic data for the structures have been deposited with the CCDC under deposition numbers 2402040 and 2402954.

**Crystal data for 1**· ***n*H**_**2**_ **O**: C_38_H_35_N_15_O_6_Pb_3_, ClO_4_, H_2_O; *M*_r_ = 1636.29, triclinic, *P*1̅, *a* = 10.8638(7) Å, *b* = 11.1056(7) Å, *c* = 21.2153(14) Å, *α* = 95.106(2)°, *β* = 100.322(2)°, *γ* = 107.175(2)°, *V* = 2378.3(3) Å^3^, *Z* = 2, *ρ* = 2.285 g cm^–3^, *μ*(Mo–Kα) = 10.789 mm^–1^, reflections: 145662 collected, 11818 unique, *R*_int_ = 0.048, *R*_1_(all) = 0.0318, *w*R**_2_(all) = 0.0622, *S* = 1.068.

**Crystal data for 2**·**H**_**2**_ **O:** C_24_H_21_N_10_O_3_Pb_2_, ClO_4_, H_2_O; *M*_r_ = 1029.35, orthorhombic, space group *Pna*2_1_, *a* = 18.6024(10), *b* = 12.0365(6), *c* = 13.1510(6) Å, *V* = 2944.6(3) Å^3^, *Z* = 4, *ρ* = 2.322 g cm^–3^, *μ*(Mo–Kα) = 11.577 mm^–1^, reflections: 175872 collected, 9297 unique, *R*_int_ = 0.082, *R*_1_(all) = 0.0439, *w*R**_2_(all) = 0.0617, *S* = 1.048.

### 2.4 DFT Calculations

Theoretical studies were carried out using the PBE0 functional, the D4 dispersion correction and the def2-TZVP basis set^88–90^ using the X-ray coordinates as implemented in the Turbomole 7.7 suite.^91^ The quantum theory of atoms in molecules (QTAIM) analysis^92^ was conducted using *Multiwfn* at the same computational level to examine topological features of the electron density.^93^ Additionally, reduced density gradient (RDG)^94^ and electron localization function (ELF)^95^ 2D plots were generated using *Multiwfn*. The QTAIM representations were visualized using *VMD* for three-dimensional visualization of the noncovalent interactions.^96^ The Laplacian can be decomposed into the eigenvalues (λ_1_, λ_2_ and λ_3_) of the Hessian matrix. The sign of λ_2_ is used for distinguishing bonding interactions (λ_2_ < 0) from closed-shell or nonbonding interactions (λ_2_ > 0), based on the sign of the second eigenvalue of the electron density Hessian.

## 3 Results and Discussion

A mixture of Pb­(ClO_4_)_2_ and KCN in water was combined with **HL** in methanol, yielding yellow needle-like crystals of a new coordination polymer {[Pb_3_L_3_(OAc)­(H_2_O)]­(ClO_4_)_2_}_*n*_·*n*H_2_O (**1**·*n*H_2_O) (Scheme 1). Interestingly, the same reaction using **NaL** produced yellow needle-like crystals of complex [Pb_2_L_2_OH]­(ClO_4_)·H_2_O (**2**·H_2_O) (Scheme 1). Notably, the isolated crystals possessed sufficient quality for structural characterization via single-crystal X-ray diffraction, eliminating the need for recrystallization. The resulting metallocomplexes were supported by the elemental analysis and their structures were further probed by FTIR and ^1^H NMR spectroscopy. Structural elucidation of the crystalline complexes was accomplished using single-crystal X-ray diffraction analysis.

**Scheme 1:**
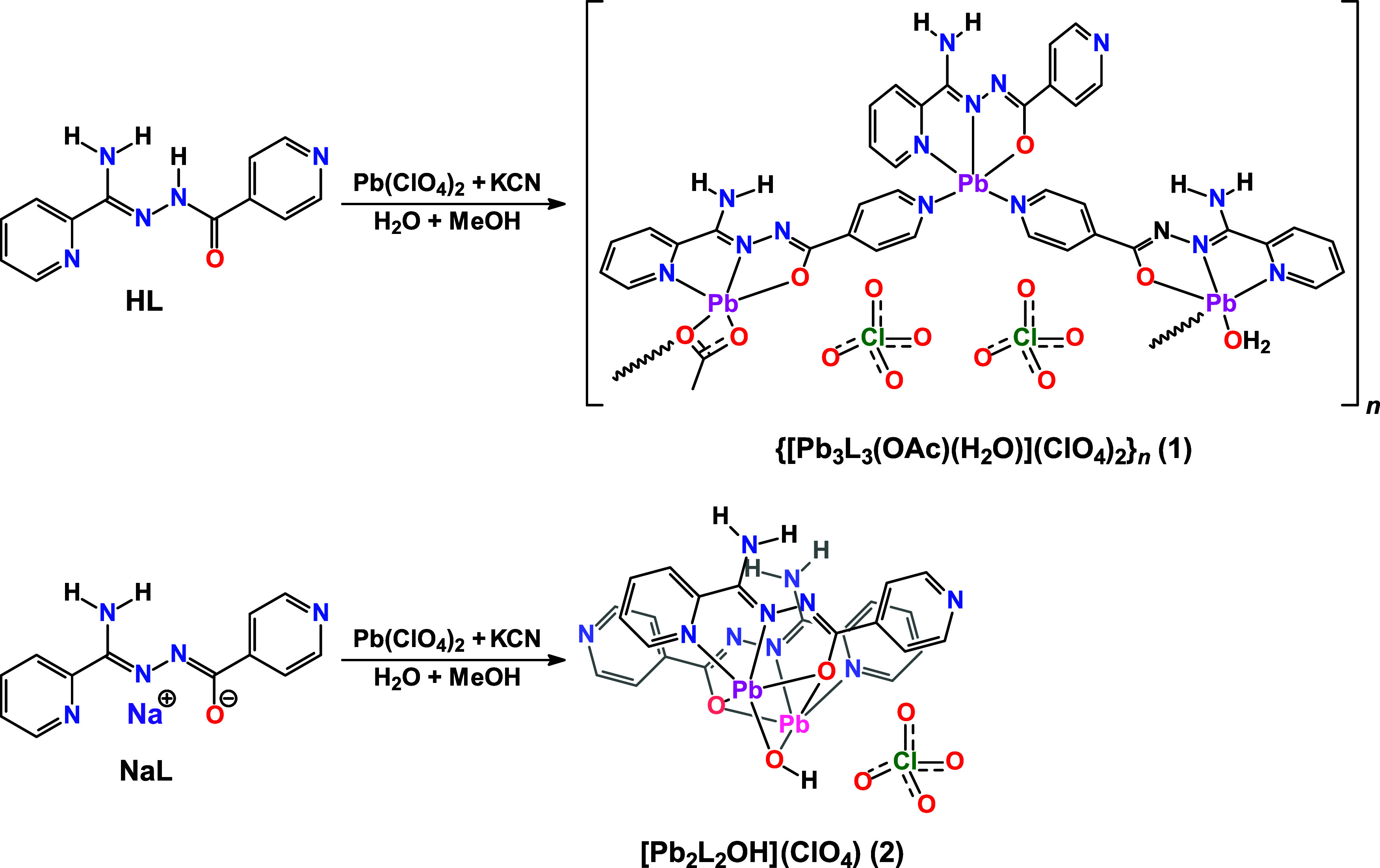
Synthetic Protocol of Complexes **1** and **2**

Intriguingly, the generation of the acetate anion likely proceeds via methanol cyanation, mediated by the presence of Pb^2+^ and cyanide under the experimental conditions. Thus, we can tentatively assign the latter salt–organic compound combination as a system, inducing the cyanation of methanol. This finding is intriguing and further in-depth studies are required, which will be initiated in due course and the obtained results will be published elsewhere.

The FTIR spectra of the obtained metallocomplexes exhibit a broad absorption in the 3000–3200 cm^–1^ range, associated primarily with C–H stretching vibrations of the aromatic moieties and contributions from acetate species in the spectrum of **1**·*n*H_2_O (Figure 1). Three bands at 3340–3400, 3450–3520 and 3615–3640 correspond to the NH_2_ vibrations and H_2_O species, and also to the OH group in **2**·H_2_O, while an intense broad band at ∼1075–1080 cm^–1^ corresponds to perchlorate (Figure 1). The ^1^H NMR spectra of the complexes display resonances consistent with the parent organic ligand, reflecting preservation of its structural features upon coordination. In particular, the 2-pyridine fragment was shown as triplet signals observed at 7.73 and 8.19 ppm, along with doublets centered at 8.29 and 8.85 ppm in the spectrum of **1**·*n*H_2_O, wile the same fragment in the spectrum of **2**·H_2_O was shown as a broad triplet at 8.13 ppm, two broad doublets at 8.28 and 8.71 ppm, and a broad singlet at 7.64 ppm (Figure 1). The high-field triplet is significantly overlapped with a broad singlet from the NH_2_ protons in the spectra of both complexes (Figure 1). The 4-pyridine fragment was revealed as two doublets or as two broad singlets at 8.01–8.11 and 8.56–8.61 ppm (Figure 1). Furthermore, the ^1^H NMR spectrum of **1**·*n*H_2_O also exhibits a singlet at 1.52 ppm, which corresponds to the acetate CH_3_ protons (Figure 1). Notably, all the signals in the ^1^H NMR spectra of the discussed complexes either were shown with the similar chemical shifts or are somewhat downfield shifted relative to the chemical shifts observed for the free ligand **HL** under identical solvent conditions.^84^

**Figure 1:**
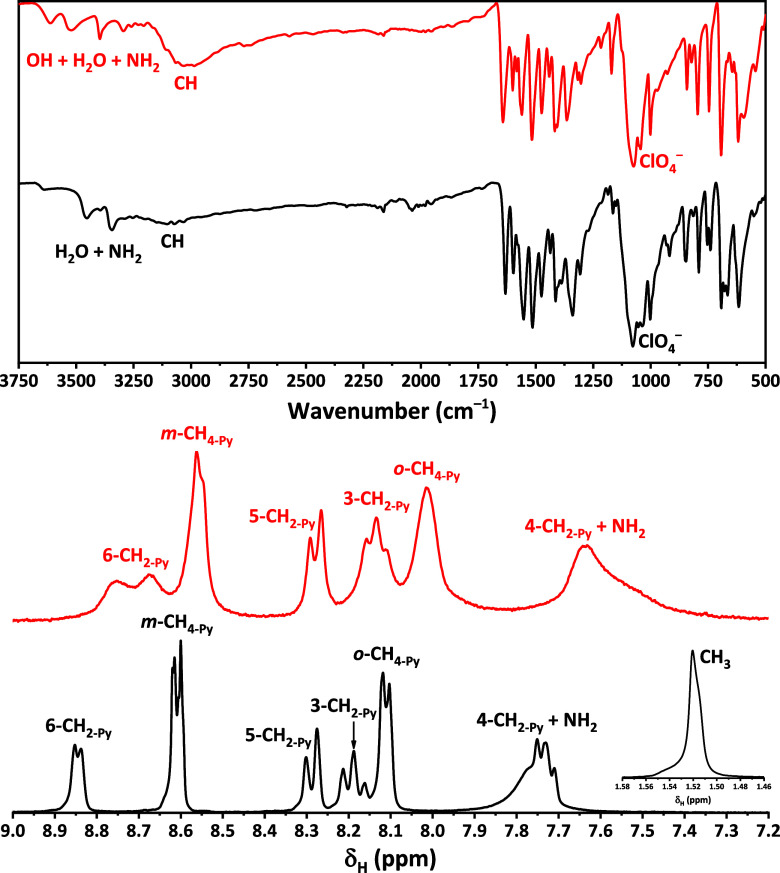
FTIR (top) and ^1^H NMR (bottom) spectra of **1**·*n*H_2_O ( black) and **2**·H_2_O (red).

A comparative analysis of the FTIR and ^1^H NMR spectra of the complexes and the parent ligand **HL** ^92^ supports the formation of the metallocomplex.

The crystal structure of **1**·*n*H_2_O was determined to crystallize in the triclinic space group *P*1̅. The asymmetric unit comprises three Pb^2+^ centers (Pb1, Pb2 and Pb3) and three anionic ligands L coordinated to the metal ions, one coordinated acetate anion, one coordinated water molecule, two perchlorate anions and one crystal water molecule. Each Pb^2+^ center is tridentately coordinated by ligand L via the 2-pyridyl and imine nitrogen atoms, along with the oxygen atom from the carbonyl group (Figure 2). The Pb–N_2‑Py_, Pb–N_imine_ and Pb–O_carbonyl_ bonds are similar within each [PbL]^+^ cation and of about 2.60–2.62, 2.37–2.40 and 2.35–2.38 Å, respectively (Table 1). Notably, the Pb–N_imine_ and Pb–O_carbonyl_ bond distances are comparable, whereas the Pb–N_2‑py_ bonds are markedly elongated (Table 1).

**Figure 2:**
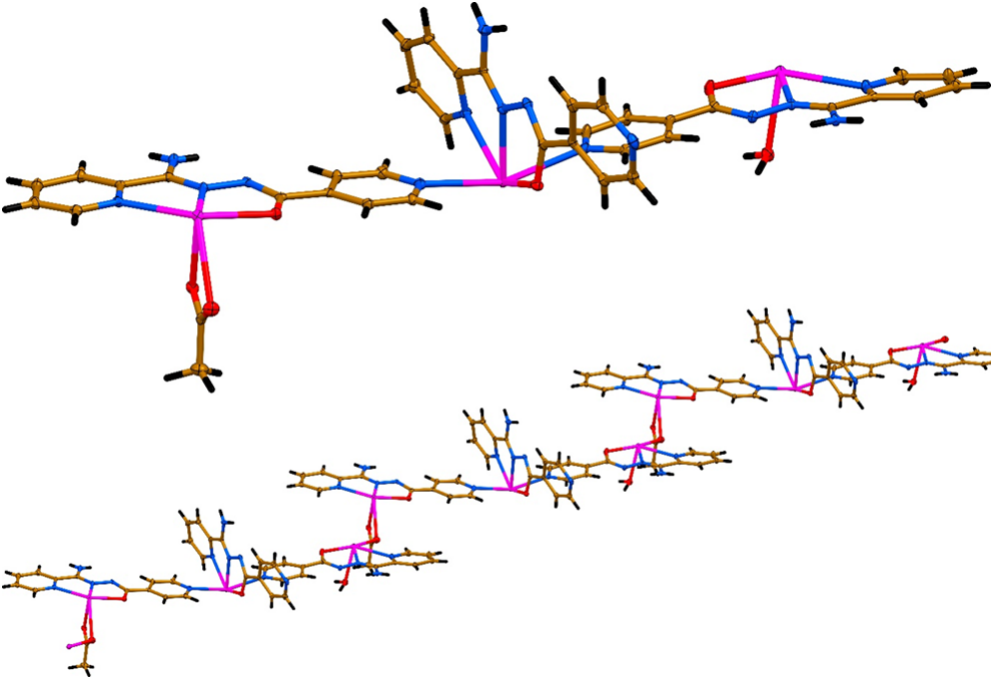
A symmetric unit (top) and 1D polymeric chain (bottom) in the crystal structure of **1**·*n*H_2_O (ClO_4_^–^ anions and the crystal water molecules are not shown for the sake of clarity). Color code: hydrogen atoms in black, carbon atoms in gold, nitrogen atoms in blue, oxygen atoms in red, chlorine atoms in green and lead atoms in magenta.

**Table 1:** Bond Lengths (Å) in the Crystal Structures of **1** and **2** with Indication of the Type of Bond

**bond**	**length**	**type**	**bond**	**length**	**type**
Complex **1**·*n*H_2_O^a^					
Pb1–N1	2.603(4)	covalent	Pb2–N7	2.370(5)	covalent
Pb1–N2	2.402(4)	covalent	Pb2–N14	2.645(4)	covalent
Pb1–O1	2.377(4)	covalent	Pb2–O2	2.353(3)	covalent
Pb1–O1W	2.476(4)	covalent	Pb2···Cg^#4^	2.92	tetrel
Pb1–O5^#1^	2.869(4)	covalent	Pb3–N11	2.606(4)	covalent
Pb1···O11^#2^	2.933(4)	tetrel	Pb3–N12	2.372(3)	covalent
Pb1···O12^#2^	3.331(4)	tetrel	Pb3–O3	2.375(3)	covalent
Pb1···O21^#3^	3.189(4)	tetrel	Pb3–O4	2.375(3)	covalent
Pb1···O23^#3^	3.930(4)	tetrel	Pb3–O5	2.815(4)	covalent
Pb2–N4	2.612(4)	covalent	Pb3···O12^#5^	3.413(4)	tetrel
Pb2–N6	2.618(4)	covalent	Pb3···Cg^#6^	3.06	tetrel
Complex **2**·H_2_O^b^					
Pb1–N1	2.665(7)	covalent	Pb2–N6	2.667(7)	covalent
Pb1–N2	2.305(7)	covalent	Pb2–N7	2.320(7)	covalent
Pb1···N8^#1^	3.230(9)	tetrel	Pb2···N3^#4^	3.198(9)	tetrel
Pb1–O1	2.347(6)	covalent	Pb1–O2	2.359(6)	covalent
Pb1–O3	2.270(10)	covalent	Pb1–O3	2.283(10)	covalent
Pb1···O11^#2^	3.438(11)	tetrel	Pb1···O12^#4^	3.468(9)	tetrel
Pb1···O1W^#3^	3.563(10)	tetrel			

a Symmetry codes: #1: *x*, −1 + *y*, −1 + *z*; #2: −1 + *x*, −1 + *y*, *z*; #3:1 – *x*, −*y*, 1 – *z*; #4:1 – *x*, 1 – *y*, 2 – *z*; #5: −1 + *x*, *y*, 1 + *z*; #6 – *x*, 1 – *y*, 3 – *z*. Cg = **L**_H33–C33–C32–C31–N13–N12–C30–N15–H15B_···O14_perchlorate_.

b Symmetry code: #1 1 – *x*, 1 – *y*, 1/2 + *z*; #2 *x*, −1 + *y*, *z*; #3 3/2 – *x*, −1/2 + *y*, 1/2 + *z*; #4 1 – *x*, 1 – *y*, −1/2 + *z*.

The coordination environments of Pb1 and Pb3 are completed by a water molecule (for Pb1) and two oxygen atoms from the acetate ligand (for Pb3), respectively (Figure 2). The Pb1–O_water_ bond length is about 2.48 Å, while, notably, the two Pb3–O_acetate_ bonds differ significantly and of about 2.38 and 2.82 Å (Table 1). Notably, the almost linear trinuclear cationic species [Pb_3_L_3_(OAc)­(H_2_O)]^2+^, which constitutes an asymmetric unit, is formed since the Pb2 cation of the central [PbL]^+^ moiety is additionally coordinated by the 4-pyridine nitrogen atoms of the other two [PbL]^+^ fragments with the similar Pb2–N_4‑Py_ bonds of about 2.61 and 2.65 Å (Table 1, Figure 2). The intramolecular Pb1···Pb2 and Pb2···Pb3 distances within the [Pb_3_L_3_(OAc)­(H_2_O)]^2+^ unit measure approximately 9.82 and 9.98 Å, with a Pb1···Pb2···Pb3 angle close to 166.4°. The electroneutrality of this trinuclear cationic species is assured by two perchlorate anions. Trinuclear cations [Pb_3_L_3_(OAc)­(H_2_O)]^2+^ are linked into a 1D cationic coordination polymer through one of the oxygen atoms of an acetate ligand, adopting a μ-bridging coordination mode between adjacent lead centers (Figure 2). This acetate oxygen is additionally coordinated to the Pb1 cation with the corresponding bond length of about 2.87 Å (Table 1).

Thus, the Pb1, Pb2 and Pb3 cations form five covalent bonds, which revealed a N_2_O_3_ for Pb1 and Pb3, and a N_4_O for Pb2 coordination environments (Figures 2 and 3). These covalent bonds are collected on an almost quarter (for Pb1 and Pb3) or on an almost half (for Pb2) the spatial distribution of donor atoms resulting in a significant open sector within the coordination sphere of the metal centers (Figure 2). This gap allows the four oxygen atoms from two perchlorate anions to closely interact with the Pb1 center with the formation of four Pb···O tetrel bonds of about 2.93, 3.19, 3.33 and 3.93 Å (Figure 3, Table 1). Interestingly, for the Pb2 and Pb3 metal cations, this coordination gap facilitates the proximity of the ten-membered hydrogen-bonded supramolecular ring Cg with the *R*_2_^1^(10) graph set (Figure 3, Table 2). These rings are essentially planar with the deviations of the involved atoms varying from about 0.01 Å to 0.14 Å. Furthermore, these rings were found to be quasi-aromatic, as indicated by HOMHED values of 0.741 and 0.729.^97^ The Pb2···Cg and Pb3···Cg tetrel bonds between the metal cations and the center of gravity of these rings are about 2.92 and 3.06 Å, respectively (Table 1). Moreover, the Pb3 cation further forms an additional Pb···O tetrel bond with the second oxygen atom of the same ClO_4_^–^ unit, which participates in the formation of the supramolecular Cg ring (Figure 3, Table 1). Taking into account both covalent and tetrel interactions, the coordination environments of the Pb1, Pb2 and Pb3 centers can be described as N_2_O_7_, N_4_OCg and N_2_O_4_Cg, respectively (Figure 3).

**Figure 3:**
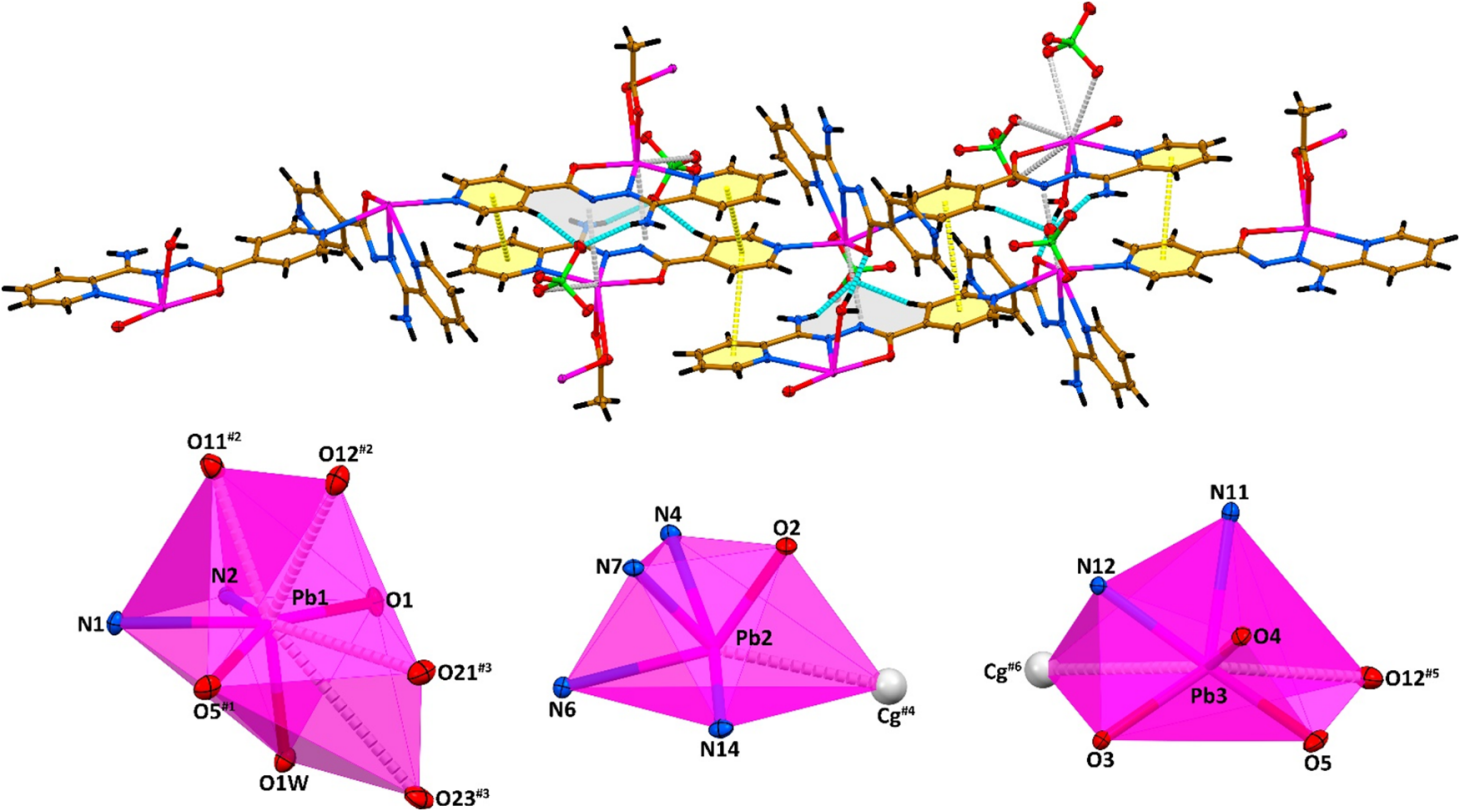
(top) Supramolecular aggregate in the crystal structure of **1**·*n*H_2_O (Cg = **L**_H33–C33–C32–C31–N13–N12–C30–N15–H15B_···O14_perchlorate_). (bottom) The coordination polyhedra are shown. Color code as in Figure 2.

**Table 2:** Hydrogen Bond Lengths (Å) and Angles (°) in the Crystal Structure of Complexes

**D–H**···**A**	***d*** **(D–H)**	***d*** **(H**···**A)**	***d*** **(D**···**A)**	**∠(DHA)**	**symmetry operation**
Complex **1**·*n*H_2_O					
O1W–H1A···O2	0.93	1.85	2.778(5)	169	1 – *x*, 1 – *y*, −*z*
O1W–H1B···O23	0.97	1.86	2.796(5)	161	1 – *x*, – *y*, −*z*
O2W–H2A···O13	0.91	2.38	3.283(9)	172	–*x*, 1 – *y*, −*z*
O2W–H2B···O3	0.87	2.05	2.895(7)	165	*x*, *y*, *z*
N5–H5A···O4	0.84(6)	2.06(6)	2.874(5)	162(6)	*x*, *y*, −1 + *z*
N5–H5B···O24	0.74(6)	2.44(6)	3.104(6)	152(6)	1 – *x*, 1 – *y*, −*z*
N10–H10B···O22	0.83(6)	2.25(7)	3.020(7)	156(6)	–1 + *x*, *y*, *z*
N15–H15A···O11	0.79(6)	2.28(6)	3.050(6)	165(7)	*x*, *y*, 1 + *z*
N15–H15B···O14	0.70(6)	2.36(6)	3.023(6)	160(7)	–*x*, −*y*, −*z*
C13–H13···O23	0.95	2.24	3.155(7)	161	*x*, *y*, *z*
C15–H15···O1	0.95	2.33	3.184(7)	149	–*x*, −*y*, −*z*
C16–H16···O22	0.95	2.59	3.518(7)	166	–1 + *x*, *y*, *z*
C22–H22···N9	0.95	2.59	3.451(8)	151	–*x*, 2 −*y*, −*z*
C25–H25···O21	0.95	2.58	3.272(7)	130	1 – *x*, 1 – *y*, 1 – *z*
C28–H28···O11	0.95	2.39	3.306(7)	162	*x*, *y*, 1 + *z*
C28–H28···O13	0.95	2.43	3.198(7)	137	*x*, *y*, 1 + *z*
C33–H33···O14	0.95	2.48	3.417(7)	169	–*x*, −*y*, −*z*
C35–H35···O1W	0.95	2.56	3.480(6)	163	1 – *x*, 1 – *y*, −*z*
C36–H36···O2W	0.95	2.55	3.474(9)	165	*x*, *y*, *z*
Complex **2**·H_2_O					
O1W–H1A···O14	0.86	2.26	3.07(2)	157	1/2 + *x*, 3/2 – *y*, *z*
O1W–H1B···O11	0.87	2.43	3.27(2)	163	3/2 – *x*, – 1/2 + *y*, −1/2 + *z*
O3–H3A···O14	0.72(10)	2.32(10)	3.020(18)	165(1)	*x*, – 1 + *y*, *z*
N5–H5A···O12	0.87(11)	2.38(11)	3.161(10)	149(10)	*x*, *y*, *z*
N5–H5B···N4	0.88(11)	2.04(11)	2.909(10)	168(10)	1/2 + *x*, 3/2 – *y*, *z*
N10–H10A···O1W	0.93(12)	2.50(11)	3.344(14)	151(9)	–1/2 + *x*, 3/2 – *y*, *z*
N10–H10B···N9	0.93(11)	1.98(11)	2.882(11)	164(9)	–1/2 + *x*, 3/2 – *y*, *z*

The Pb···Cg and Pb···O tetrel bonds link 1D cationic coordination polymers into a 3D supramolecular framework, which is further reinforced by a myriad of hydrogen bonds (O–H···O, N–H···O and C–H···O) (Table 2), and π-stacking interactions (Table 3) between the pyridine moieties (Figures 3 and 4).

**Figure 4:**
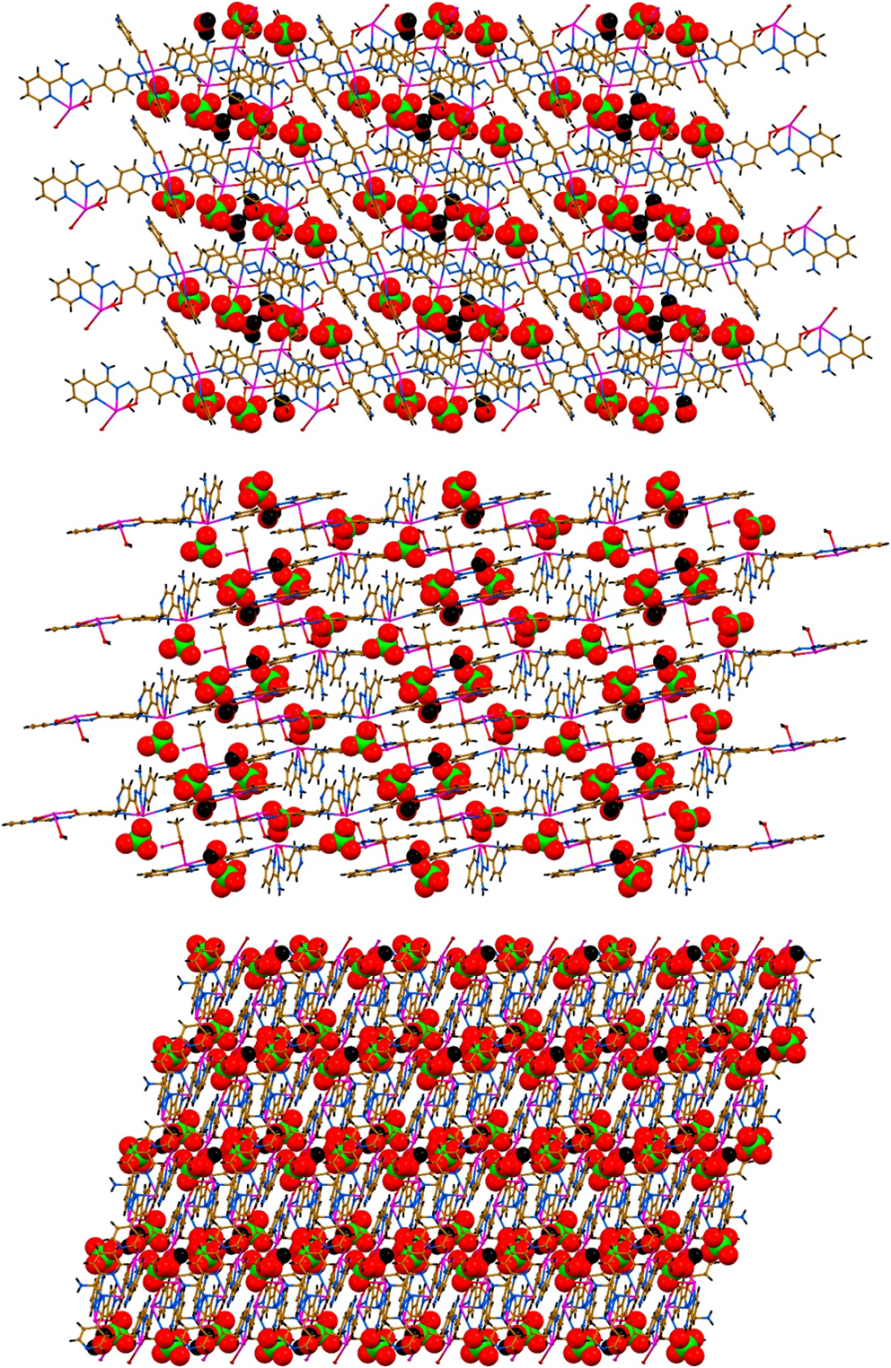
Crystal packing of **1**·*n*H_2_O along the *a* axis (top), *b* axis (middle) and *c* axis (bottom).

**Table 3:** π···π-Stacking Interaction Distances (Å) and Angles (°) in the Crystal Structures of Complexes

**Cg(** ***I*** **)**	**Cg(** ***J*** **)**	***d*** **[Cg(*****I*****)**···**Cg(** ***J*** **)]**	**α**	**β**	**γ**	**slippage**	**symmetry operation**
Complex **1**·*n*H_2_O							
2-Py_N1_	4-Py_N14_	3.776(3)	10.1(3)	22.3	26.8	1.434	1 – *x*, 1 – *y*, −*z*
4-Py_N14_	2-Py_N1_	3.776(3)	10.1(3)	26.8	22.3	1.704	1 – *x*, 1 – *y*, −*z*
2-Py_N11_	4-Py_N14_	3.590(3)	4.8(3)	20.4	23.0	1.252	–*x*, 1 – *y*, 1 – *z*
4-Py_N14_	2-Py_N11_	3.590(3)	4.8(3)	23.0	20.4	1.405	–*x*, 1 – *y*, 1 – *z*
4-Py_N4_	4-Py_N4_	4.074(3)	0.0(3)	33.1	33.1	2.226	1 – *x*, 1 – *y*, −*z*
Complex **2**·H_2_O							
2-Py_N1_	2-Py_N6_	3.942(6)	2.3(5)	31.7	31.9	2.071	1/2 + *x*, 1/2 – *y*, *z*
2-Py_N6_	2-Py_N1_	3.942(6)	2.3(5)	31.9	31.7	2.086	–1/2 + *x*, 1/2 – *y*, *z*

Complex **2**·H_2_O crystallizes in the orthorhombic *Pna2*_*1*_ space group, with the asymmetric unit comprising one [Pb_2_L_2_OH]^+^ cation, one perchlorate anion and one crystal water molecule. The structure of the mentioned cation can be described as a discrete dinulear heteroleptic dimer, where two mononuclear cations [PbL]^+^ are face-to-tail bridged by the hydroxide anion, which is μ-coordinated to both Pb^2+^ metal cations. These two [PbL]^+^ fragments within the [Pb_2_L_2_OH]^+^ cation are rotated relative to each other with a N2–Pb1···Pb2–N7 torsion angle of ∼42.6° and a Pb1···Pb2 separation of 4.3656(4) Å (Figure 5). Two [PbL]^+^ species are essentially planar, although the one formed by the Pb1 cation is slightly more planar in comparison to the Pb2-based one, and their mean planes are ∼11.2° tilted to each other. The greatest deviations from the mean plane of the [PbL]^+^ cation were revealed for the metal cation (0.237 and 0.253 Å for [Pb1L]^+^ and [Pb2L]^+^, respectively), amine nitrogen atom (0.147 and 0.192 Å for [Pb1L]^+^ and [Pb2L]^+^, respectively) and carbonyl oxygen atom (0.122 and 0.109 Å for [Pb1L]^+^ and [Pb2L]^+^, respectively). Furthermore, the amide nitrogen atom and the 2-pyridine carbon atom in the [Pb2L]^+^ fragment are also >0.1 Å deviated from the mean plane with the values of 0.103 and 0.108 Å, respectively. Other atoms in both [PbL]^+^ cations are only 0.002–0.093 Å deviated from the mean plane.

**Figure 5:**
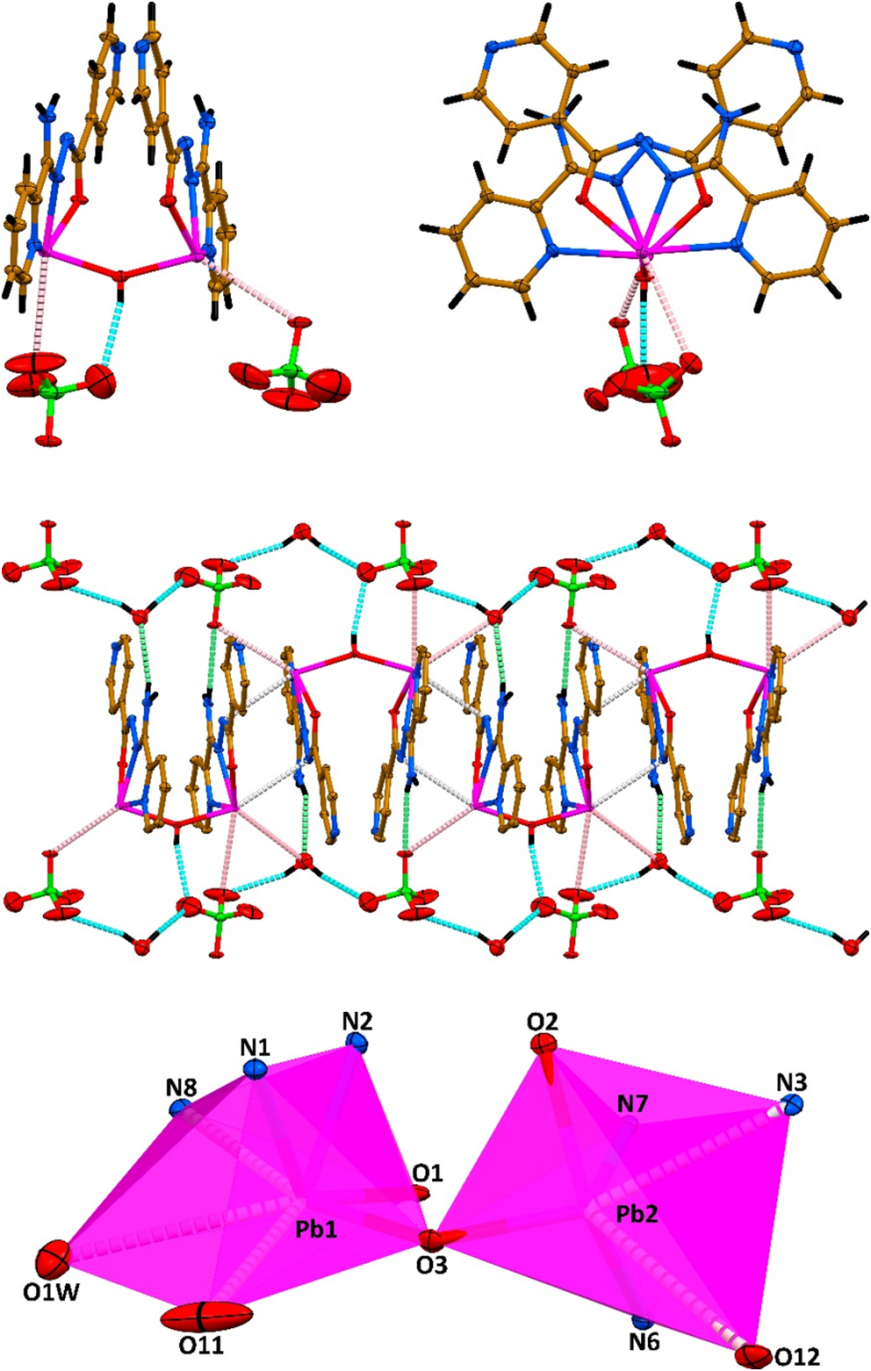
(top) Different views of the {[Pb_2_L_2_OH]_2_(ClO_4_)_2_}^−^ unit in the crystal structure of **2**·H_2_O. (middle) A 1D supramolecular chain constructed through the Pb···N and Pb···O tetrel bonds, and N–H···O and O–H···O hydrogen bondsin the crystal structure of **2**. (bottom) The coordination polyhedra are shown. Color code as in Figure 2.

In complex **2**·H_2_O, the ligand L binds to Pb^2+^ via the 2-pyridyl and imine nitrogen atoms as well as the carbonyl oxygen, generating two five-membered chelate rings (Figure 5). The Pb–N_imine_ bonds measure approximately 2.31–2.32 Å, whereas the Pb–N_2__‑pyridyl_ separations are about 0.3 Å longer (Table 1). The Pb–N_imine_ and Pb–O_carbonyl_ distances are similar and of about 2.35–2.36 Å (Table 1). The Pb–O bonds formed with the bridging hydroxide anion are ∼2.27–2.28 Å with a Pb1–O3–Pb2 bond angle of 147.0(2)° (Table 1). Thus, the metal cations form four covalent bonds, which revealed a N_2_O_2_ coordination environment (Figure 5). These covalent bonds are arranged over nearly one-quarter of the coordination space, leaving a significant open sector in the Pb^2+^ coordination sphere, which facilitates the approximation of the amide N atom belonging to an adjacent [Pb_2_L_2_OH]^+^ cation resulting in tetrel-type interactions of ∼3.2 Å (Figure 5, Table 1). These contacts promote the formation of a 1D polymer exhibiting a zig-zag topology (Figure 5).

The Pb^2+^ coordination environments are completed by interactions with oxygen atoms from perchlorate anions, with Pb···O contacts of ∼3.44–3.47 Å (Figure 5, Table 1). Finally, the O atom of the crystal water molecule completes the coordination of the Pb1 cation with the Pb1···O separation of ∼3.56 Å (Figure 5, Table 1). Interestingly, all these Pb···O interactions with the perchlorate anions and a water molecule are considered as tetrel bonds. Including both covalent and tetrel-type interactions, the coordination geometries of Pb1 and Pb2 correspond to N_3_O_4_ and N_3_O_6_ arrangements, respectively (Figure 5).

Notably, one perchlorate oxygen, coordinated to Pb1, engages in an O–H···O hydrogen bond with the hydroxide group. Additionally, the perchlorate bound to Pb2 participates in N–H···O hydrogen bonding with an NH_2_ hydrogen from a neighboring [Pb_2_L_2_OH]^+^ unit (Figure 5, Table 2). Furthermore, the water oxygen atom also forms a hydrogen bond with one of the NH_2_ hydrogen atoms, while each H atom forms a O–H···O hydrogen bond with the O atoms belonging to the neighboring perchlorate anions (Figure 5, Table 2). Thus, all these hydrogen bonds further strengthen and link 1D supramolecular polymeric cationic chains, yielding a 2D supramolecular sheets (Figure 6). Interlayer π···π stacking between 2-pyridyl moieties connects these 2D supramolecular sheets into an extended 3D framework (Table 3, Figure 6).

**Figure 6:**
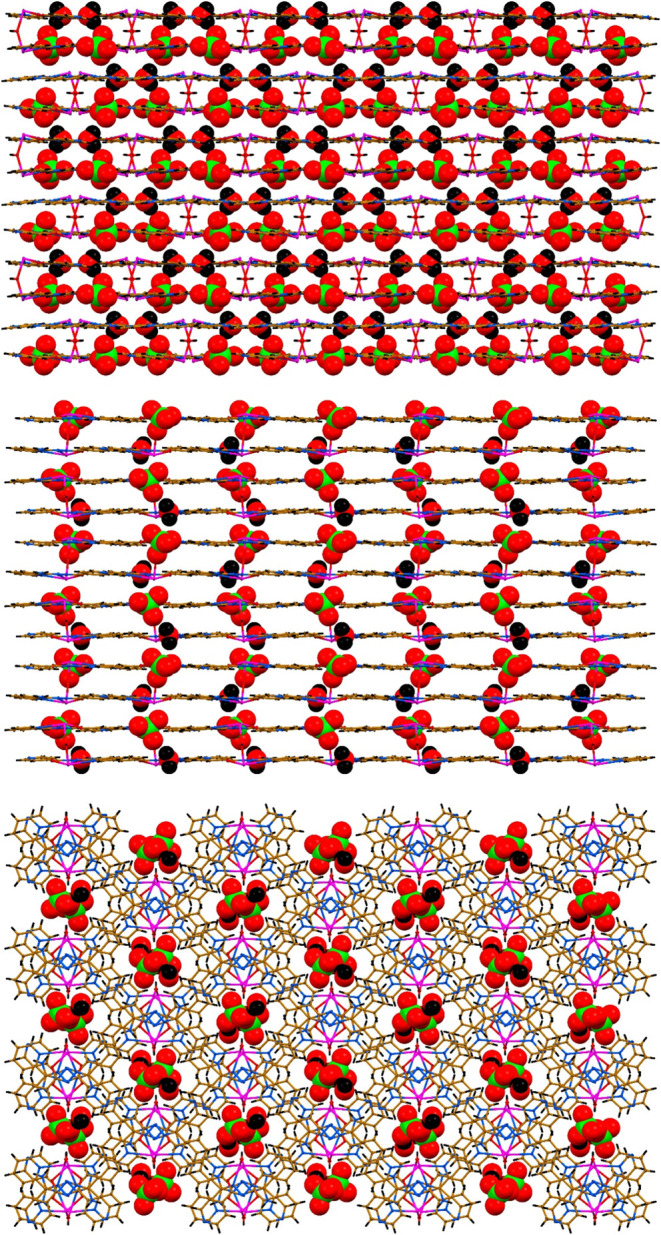
Crystal packing of **2**·H_2_O along the *a* (top), *b* (middle) and *c* (bottom) axes.

The optical properties of both complexes were studied in methanol using UV–vis spectroscopy and photoluminescence. It was found that both complexes possess very similar optical properties in MeOH. In particular, complexes exhibit absorption extending to approximately 470 nm, featuring distinct peaks near 225 and 380 nm (Figure 7). The high-energy band is accompanied by shoulders at about 255, 275 and 288 nm (Figure 7). The corresponding parent compounds **HL** and **NaL** display absorption bands reaching up to ∼430 nm, with prominent maxima at 215, 275 and 325 nm (Figure 7). These absorption features are primarily ascribed to intraligand *n* → π* and π → π* electronic transitions. Solutions of both complexes in MeOH were found to be emissive from about 420 to 750 nm when exciting at 380 nm, yielding a broad band at about 560 nm (Figure 7). This band is accompanied by a distinct shoulder near 622 nm (Figure 7). Emission band deconvolution revealed two components centered at 545 and 619 nm for **1**·*n*H_2_O, and at 549 and 621 nm for **2**·H_2_O (Figure 7). Furthermore, solutions of both **HL** and **NaL** in the same solvent were also found to be emissive but from about 400 to 700 nm when excited at 370 nm, with a broad luminescence band peaking at 508 nm (Figure 7). The same deconvolution process applied to the emission band revealed superposition of two bands with maxima at 496 and 541 nm for **HL**, and at 492 and 551 nm for **NaL** (Figure 7). Correlation of the emission, excitation and absorption spectra of the complexes with the corresponding spectra of **HL** and **NaL** enabled identification of the emission origin as arising from a combination of intraligand and ligand-to-metal charge transfer processes with some contribution from metal-centered s → p transitions in **1**·*n*H_2_O and **2**·H_2_O.^10,98–101^ The observed Stokes shift (∼180 nm) is large for both complexes and significantly superior to those of **HL** (∼130 nm) and **NaL** (∼135 nm), and has been attributed in the literature to substantial structural rearrangement between the ground and excited states, a phenomenon common in heavy-metal complexes with an s^2^ configuration, where distortion in the ground state can be relieved in the excited state, favoring low-energy metal-centered transitions and potentially enabling phosphorescent emission.^100^

**Figure 7:**
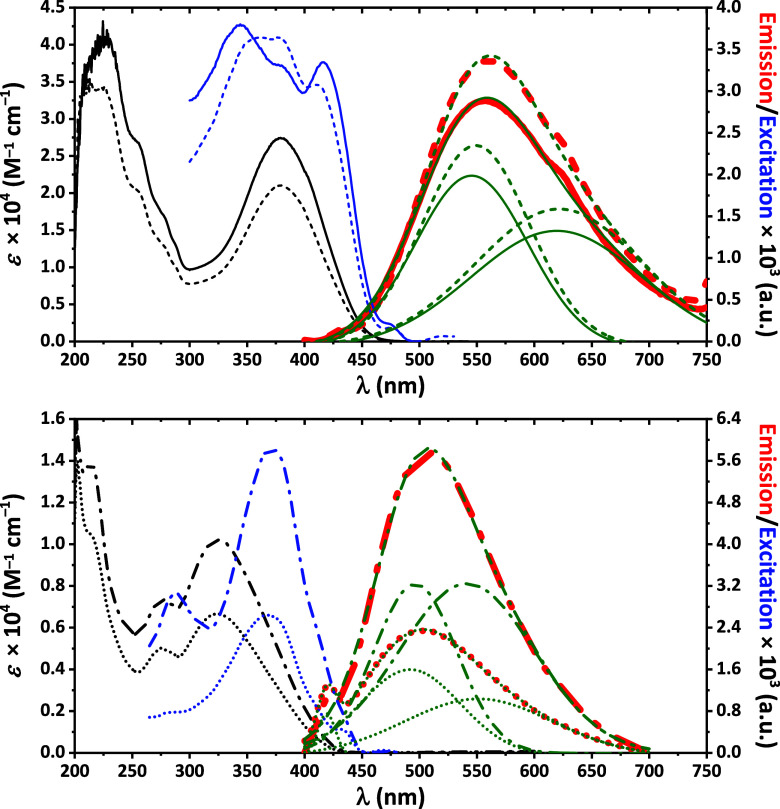
(top) The absorption (black), emission (red, λ_exc_ = 380 nm) and excitation (blue, λ_em_ = 560 nm) spectra for **1**·*n*H_2_O (solid line) and **2**·H_2_O (dashed line) in MeOH at room temperature. (bottom) The absorption (black), emission (red, λ_exc_ = 370 nm) and excitation (blue, λ_em_ = 508 nm) spectra for **HL** (dot line) and **NaL** (dash-dot line) in MeOH at room temperature. Deconvolution (green) of the emission spectra for **1**·*n*H_2_O (solid line, *R* ^2^ = 0.9970), **2**·H_2_O (dashed line, *R* ^2^ = 0.9946), **HL** (dot line, *R* ^2^ = 0.9974) and **NaL** (dash-dot line, *R* ^2^ = 0.9995) are given.

Regarding colorimetry, the CIE-1931 coordinates calculated from the emission spectra (Figure 7) were *x* = 0.409, *y* = 0.492 for **1**·*n*H_2_O and *x* = 0.420, *y* = 0.498 for **2**·H_2_O, placing both complexes in the yellow green region of the chromaticity diagram, consistent with their emission wavelengths and in agreement with similar Pb^2+^-based systems exhibiting warm or white light emission.^79,101^ The same CIE-1931 coordinates for **HL** and **NaL** were calculated as *x* = 0.256, *y* = 0.373 and *x* = 0.265, *y* = 0.419, respectively, placing both compounds in the green gamut of the chromaticity diagram.

The DFT calculations focused on analyzing the interactions that drive the formation of the supramolecular aggregate for **1** (Figure 3) as well as a monomeric fragment of the 1D supramolecular chain of **2** (Figure 5), both of which play a key role in the solid state structure of both compounds. To initiate the theoretical study, we calculated the molecular electrostatic potential surfaces for models of **1** and **2**. To better visualize the σ-holes at the Pb atoms, we used the [PbL­(OAc)] neutral fragment in **1**, while for **2**, we considered the entire ion pair [Pb_2_L_2_OH]­(ClO_4_). This approach ensures a more accurate representation of the σ-hole distribution at the Pb center.

For the [PbL­(OAc)] fragment in **1**, the molecular electrostatic potential minimum is located at the oxygen atoms of the acetate ligand, followed by the N atom of pyridine (Figure 8). The latter is coordinated to Pb2 in **1**, contributing to the extension of the coordination polymer. The molecular electrostatic potential maximum is found at the NH_2_ group (58.6 kcal/mol), consistent with its ability to form N–H···O hydrogen bonds with perchlorate ions (Table 1). The polarity of the pyridine rings in this fragment is reversed, with a positive potential over the Pb-coordinated pyridine (16.9 kcal/mol) and a negative potential over the noncoordinated pyridine (−15.1 kcal/mol), though the latter is likely reduced upon coordination to Pb2. Most importantly, the molecular electrostatic potential distribution at the Pb center is distinctly anisotropic (Figure 8), exhibiting two well-defined σ-holes. One is located opposite to the Pb–OAc bond (21.3 kcal/mol) and the other one along the extension of the Pb–O­(L) bond (29.5 kcal/mol).

**Figure 8:**
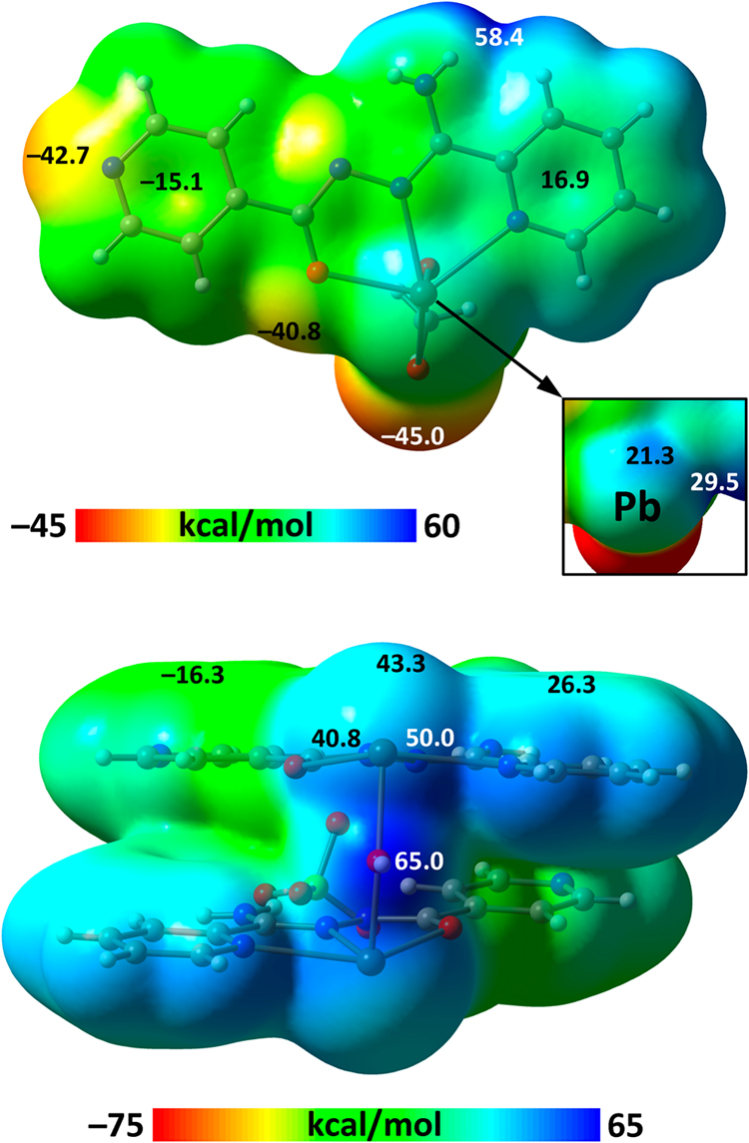
MEP surfaces of the [PbL­(OAc)] fragment of **1**·*n*H_2_O (top) and the [Pb_2_L_2_OH]­(ClO_4_) fragment of **2**·H_2_O (bottom).

For the [Pb_2_L_2_OH]­(ClO_4_) fragment in **2**, the molecular electrostatic potential maximum is found at the H atom of the bridging hydroxyl group (65 kcal/mol), while the molecular electrostatic potential minimum is associated with the perchlorate anion (not visible in the current orientation of the molecular electrostatic potential surface) (Figure 8). Like in the [PbL­(OAc)] fragment in **1**, the molecular electrostatic potential values for the pyridine rings exhibit an inverted polarity, with a positive region over the coordinated pyridine (26.3 kcal/mol) and a negative region over the noncoordinated pyridine (−16.3 kcal/mol). The Pb atom in **2** features three distinct σ-holes, one located opposite to the Pb–OH coordination bond (43.3 kcal/mol), another located opposite to the Pb–O­(L) bond (50.0 kcal/mol), and a third one along the extension of the Pb–N_Py_ coordination bond (40.8 kcal/mol) (Figure 8). These features explain the formation of multiple tetrel bonds with counterions and water molecules (Figure 5, Table 1).

In the theoretical model, used to analyze the supramolecular assembly in **1**, two monodentate pyridine rings (represented in green) are included to truncate the coordination polymer (Figure 9). This supramolecular assembly was examined using a combined QTAIM/NCI computational approach, as their synergy is particularly effective in visualizing noncovalent interactions. The analysis revealed that the [PbL­(OAc)] moieties (highlighted in pink) engage in π-stacking interactions, not only through conventional π···π interactions involving the aromatic pyridine rings but also via interactions between the five-membered chelate rings (Figure 9). A total of eight bond critical points (BCPs) interconnect the [PbL­(OAc)] moieties, confirming the presence of multiple stabilizing interactions (Figure 9). Additionally, extended reduced density gradient (RDG) isosurfaces are observed between the aromatic and chelate rings, further characterizing this binding mode (Figure 9).

**Figure 9:**
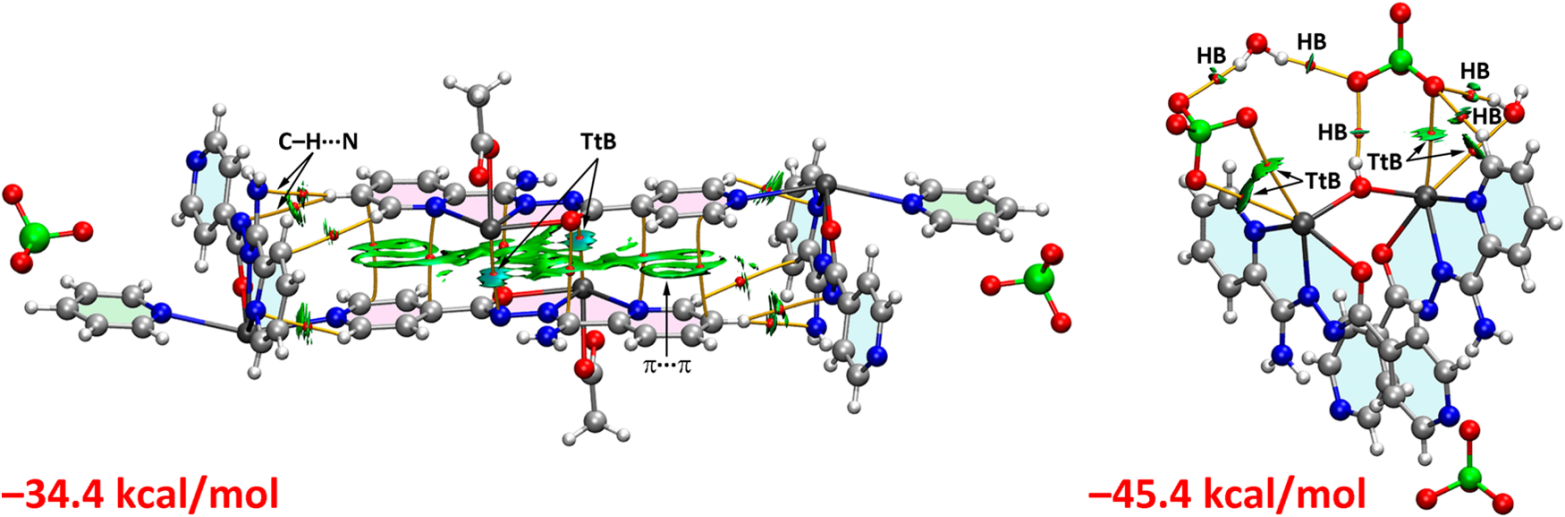
Combined QTAIM/NCI analysis for **1**·*n*H_2_O (left) and **2**·H_2_O (right). Formation energies of the assemblies are also indicated.

A particularly noteworthy observation is that two of these BCPs connect the Pb atoms with the N atoms of the hydrazido groups, Pb3···N13 contacts of 3.157(4) Å. The corresponding RDG isosurfaces appear bluish, indicating that these interactions are moderately strong (Figure 9). This finding agrees with the molecular electrostatic potential analysis of the [PbL­(OAc)] fragment, which highlighted the presence of σ-holes at the Pb center (Figure 8). Furthermore, the QTAIM/NCI analysis shows the existence of C–H···N contacts involving pyridine hydrogen atoms of one ligand and nitrogen atoms of an adjacent ligand, further reinforcing the formation of the 1D polymer in **1**. The computed formation energy of this assembly is significantly negative (−34.4 kcal/mol), underscoring the intricate interplay of noncovalent interactions and confirming their crucial role in the crystal packing of **1**.

The combined QTAIM/NCI analysis of **2** revealed a network of BCPs and bond paths interconnecting the [Pb_2_L_2_(OH)]^+^ fragment with the perchlorate anions and crystal water molecules (Figure 9). Each Pb atom forms two Pb···O tetrel bonds: one involving a bifurcated interaction with two oxygen atoms of the perchlorate anion, and the other involving one O atom from the perchlorate and another one from a crystal water unit (Figure 9). Additionally, the second water molecule bridges the two perchlorate anions without directly interacting with the [Pb_2_L_2_(OH)]^+^ fragment. The combined QTAIM/NCI analysis also proves the presence of a hydrogen bond between the bridging OH ligand and the perchlorate anion (Figure 9). The binding energy is significantly large (−45.4 kcal/mol), attributed to the combined contributions of the O–H···O hydrogen bond, Pb···O tetrel interactions, and the ion-pair nature of the system, which collectively enhance the stability of the supramolecular assembly.

To further investigate the Pb···N tetrel bonding interactions identified in the π-stacked assembly of **1** (Figure 9), we utilized combined 2D plots of ∇^2^ρ (Laplacian of ρ) and 2D reduced density gradient maps (Figure 10). The ∇^2^ρ plots reveal the degree of covalency in these interactions, while the reduced density gradient maps highlight noncovalent bonding, making this approach particularly effective for differentiating between covalent and noncovalent forces. Furthermore, the sign of λ_2_ (the second eigenvalue) of the Hessian within low reduced density gradient regions serves as a marker for attractive interactions.

**Figure 10:**
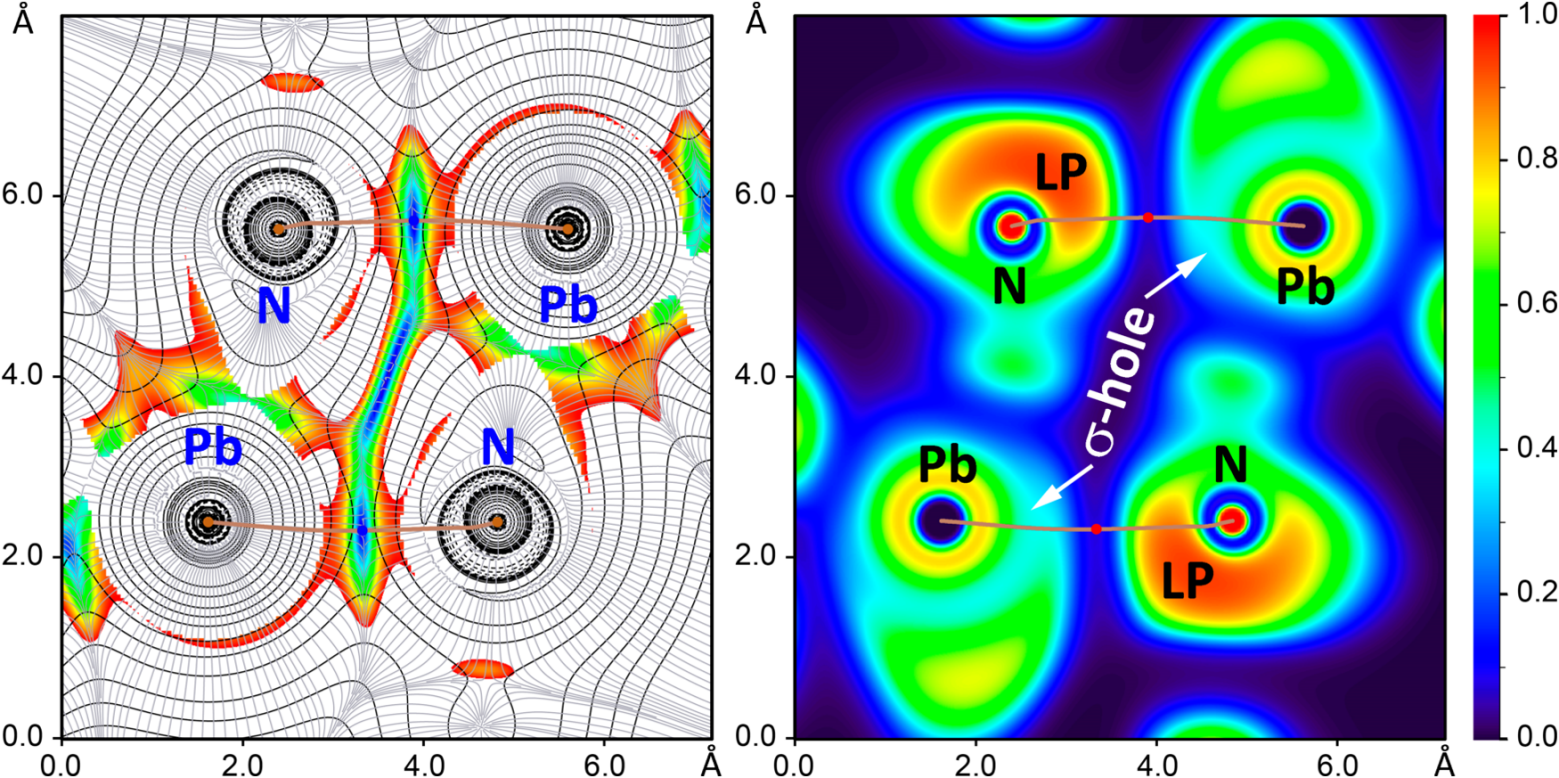
(left) Two-dimensional map plot of ∇^2^ρ combined with the reduced density gradient plot for the dimer of **1**. (right) The electron localization function (ELF) represented in a two-dimensional map of the dimer of **1**. The two-dimensional map is defined by the Pb3 and N13 atoms of both monomers.

The ELF represented in a two-dimensional map was used to visualize nucleophilic and electrophilic zones. Table 4 summarizes the BCP parameters for the Pb···N tetrel bonding interactions. The two-dimensional ∇^2^ρ plot shows positive values (solid contour lines) between the Pb and N atoms, confirming the noncovalent character of these interactions (Figure 10). This is further supported by the two-dimensional map plot of reduced density gradient, which showcase blue zones in the interaction regions. Additionally, the two-dimensional maps reveal a large region with low RDG values between the Pb···N tetrel bonds, corresponding to the π···π interaction of the chelate rings.

**Table 4:** Quantum Theory of Atoms in Molecules and Electron Localization Function Values (a.u.) for the Bond Critical Points, Characterizing the Tetrel Bonds in **1**·*n*H_2_O and **2**·H_2_O

**compound**	**tetrel bond**	**ρ(r)**	**G(r)**	**V(r)**	**H(r)**	∇^**2**^ **ρ**	**ELF**	**λ**_**2**_
**1**·*n*H_2_O	Pb3···N13	0.0142	0.0084	–0.0077	0.0007	0.0360	0.0750	–0.0106
	Pb1···O11	0.0065	0.0042	–0.0031	0.0012	0.0217	0.0233	–0.0042
	Pb1···O1W	0.0056	0.0035	–0.0024	0.0010	0.0180	0.0204	–0.0035
**2**·H_2_O	Pb2···O12	0.0068	0.0041	–0.0030	0.0011	0.0211	0.0285	–0.0045
	Pb2···O13	0.0040	0.0023	–0.0016	0.0007	0.0124	0.0145	–0.0022

The bond critical points (in red) and bond paths (in brown) are highlighted on the two-dimensional maps (Figure 10), with regions where the RDG values approach zero. Additionally, the ELF 2D map more clearly reveals the σ-hole characteristics associated with the Pb···N tetrel bonds, displaying a clear electron localization region around the N atoms pointing toward the Pb atoms. Moreover, the ELF analysis highlights the electrophilic character of the Pb atom and reveals the occurrence of a σ-hole (Figure 10). Interestingly, the bond path between the Pb and N atoms passes through the σ-hole, avoiding the inert lone pair at the Pb center (marked by the yellow ELF isocontour).

The observed low values of electron density (ρ­(r)), potential energy density (V­(r)), and Lagrangian kinetic energy density (G­(r)), together with a positive total energy density (H­(r)) and a low ELF value of 0.0750 a.u., are indicative of weak, noncovalent interactions (Table 4). The negative λ_2_ value confirms the attractive nature of the Pb···N contacts. Notably, the parameters associated with the Pb···N interactions are greater than those for the Pb···O tetrel bonds for **1** and **2** (Figure 9, Table 4). This observation is consistent with the RDG isosurfaces, which appear blue for Pb···N and green for Pb···O, confirming the relatively stronger nature of the Pb···N contacts, also supported by the shorter experimental bond distances.

## 4 Conclusions

In this work, we demonstrate that reacting a mixture of Pb­(ClO_4_)_2_ and KCN in aqueous solution with a methanolic solution of *N′*-isonicotinoylpicolinohydrazonamide (**HL**) results in the formation of a new coordination polymer, {[Pb_3_L_3_(OAc)­(H_2_O)]­(ClO_4_)_2_}*_n_* ·*n*H_2_O (**1**·*n*H_2_O), which crystallizes as yellow needle-like solids. Despite the presence of multiple starting anions (ClO_4_^–^ and CN^–^), only the acetate anion participates in the structure, likely formed in situ via methanol cyanation and subsequent oxidation. This highlights the Pb­(ClO_4_)_2_–**HL** pair as a promising system for promoting alcohol cyanation under mild conditions. A related salt-like complex, [Pb_2_L_2_OH]­(ClO_4_)·H_2_O (**2**·H_2_O), was obtained by reacting Pb­(ClO_4_)_2_ and KCN with the sodium salt of the ligand (**NaL**) under similar conditions. Again, the CN^–^ anion is absent in the final structure, while the hydroxide ion is retained and incorporated.

In both cases, the ligand participates exclusively in its deprotonated form (L), coordinating through the 2-pyridyl and imine nitrogen atoms, and the carbonyl oxygen atom, yielding a mononuclear complex cation [PbL]+ as the main building unit. In **1**·*n*H_2_O, three [PbL]^+^ units are assembled into an almost linear trimeric aggregate, stabilized by pyridine–lead bridging. The coordination environment around the terminal Pb^2+^ cations is completed by two acetate oxygen atoms or one water oxygen atom, giving rise to a secondary building unit {[Pb_3_L_3_(OAc)­(H_2_O)]^2+^}. These units assemble via covalent bonds covering about one-quarter to one-half of the coordination sphere of each metal center, leaving significant vacant space. This allows the formation of Pb···O tetrel bonds involving perchlorate anions, which, for one Pb^2+^ ion, include up to four such interactions. The remaining Pb^2+^ centers are involved in Pb···Cg tetrel bonding, where Cg is the centroid of a quasi-aromatic, ten-membered supramolecular ring stabilized by hydrogen bonding, adopting an *R*_2_^1^(10) motif. In **2**·H_2_O, two [PbL]^+^ units are bridged by a hydroxide ion in a μ-mode, forming a dinuclear cation [Pb_2_L_2_OH]^+^. Each Pb^2+^ ion in this structure is further coordinated by a perchlorate oxygen atom and one water molecule. The reciprocal Pb···N tetrel interactions link the dimers into a one-dimensional zig-zag cationic chain. These chains are then organized into 2D supramolecular sheets via N–H···O and O–H···O hydrogen bonds, which further assemble into a 3D framework through π···π stacking interactions between adjacent 2-pyridyl rings.

Theoretical calculations revealed the importance of tetrel bonding in shaping the supramolecular assemblies of the Pb^2+^ complexes. The MEP analysis evidenced the presence of distinct σ-holes at the Pb centers, supporting the formation of directional Pb···O and Pb···Ninteractions that contribute to extended architectures. QTAIM and NCI analyses confirmed the cooperative role of these tetrel bonds, which act in concert with π-stacking and hydrogen bonding to stabilize the overall solid-state organization. Calculated formation energies highlighted the significant stabilizing effect of these noncovalent forces, especially in **1**, where π-stacking between chelate rings and Pb···N contacts dominate, and in **2**, where bifurcated Pb···O tetrel bonds and hydrogen bonding collectively support the supramolecular structure.

Both complexes show photoluminescence in methanol, with broad emission bands ranging from ∼420 to 750 nm. The corresponding CIE-1931 coordinates, (0.406, 0.492) for **1**·*n*H_2_O and (0.420, 0.498) for **2**·H_2_O, fall within the yellow-green zone of the chromaticity diagram, confirming their performance as warm-emitting single-component luminophores.

To conclude, as coordination chemistry and crystal engineering continue to investigate main and auxiliary ligand-driven design of metal–organic assemblies, the insights gained here offer valuable guidance for the development of new luminescent materials.
